# sRAGE and early signs of cardiac target organ damage in mild hypertensives

**DOI:** 10.1186/s12933-019-0821-5

**Published:** 2019-02-12

**Authors:** Andrea Maria Maresca, Luigina Guasti, Sara Bozzini, Christian Mongiardi, Nicolò Tandurella, Rossana Corso, Francesco G. Zerba, Alessandro Squizzato, Leonardo Campiotti, Francesco Dentali, Catherine Klersy, Anna M. Grandi, Colomba Falcone

**Affiliations:** 1 Research Center on Dyslipidemia, Department of Medicine and Surgery, University of Insubria, Varese, Italy; 2 Interdepartimental Center for Research in Molecular Medicine (CIRMC), University of Pavia, Pavia, Italy; 3 Clinical Epidemiology and Biometry, IRCCS Policlinico San Matteo, Pavia, Italy; 4 Department of Cardiology, Istituti Clinici di Pavia e Vigevano University Hospital, Pavia, Italy

**Keywords:** sRAGE, Inflammation, Oxidative stress, Mild hypertension, Cardiac target organ damage, Left ventricular mass index, Left atrial volume index

## Abstract

**Background:**

Soluble Receptor for Advanced Glycation End Products (sRAGE) may be considered a marker inversely related to inflammation and its participation has been established in patients with advanced atherosclerotic vascular diseases. However, it is still unknown whether sRAGE reduction could be early metabolic change in the first stage of hypertension and initial hypertension-associated cardiac damage. We sought to determine the sRAGE values in otherwise healthy, untreated and recently diagnosed mild hypertensives and evaluate their association with blood pressure (BP) values, metabolic parameters, and with subclinical initial signs of cardiac target organ damage (TOD).

**Methods:**

sRAGE were measured in 100 hypertensive and 100 normotensive subjects matched for age, gender and body mass index (BMI), submitted to a clinic visit and both ambulatory BP monitoring and echocardiography to determine the presence of initial cardiac TOD (presence of signs of left ventricular hypertrophy: left ventricular mass indexed for height^2.7^ (LVMi) > 48 g/m^2.7^ for men and > 44 g/m^2.7^ for women and/or increased left atrial volume 4-chamber indexed for body surface area (LAVi) > 34 ml/m^2^).

**Results:**

sRAGE levels were similar between hypertensive and normotensive subjects and were not significantly correlated with office and 24-h BPs values. However, when subgrouping the hypertensive patients in Hyp-TOD and Hyp-withoutTOD, sRAGE was found to be different among the three groups (p = 0.030), being lower in the Hyp-TOD group than the values of both Hyp-withoutTOD (p = 0.038) and normotensives (p = 0.038). In hypertensive patients sRAGE was negatively related with both LVMi (r = − 0.239, p = 0.034) and LAVi (r = − 0.315, p = 0.005) and was independently related to cardiac TOD also in multivariable analysis.

**Conclusions:**

In this population of mild hypertensives, low circulating sRAGE may be a very early marker of initial TOD, suggesting the possible participation of oxidative stress in initial cardiac changes in human hypertension.

**Electronic supplementary material:**

The online version of this article (10.1186/s12933-019-0821-5) contains supplementary material, which is available to authorized users.

## Background

The interaction between Advanced Glycation End products (AGEs) with their cell-bound receptors (RAGE) results in oxidative stress-related stimuli and may contribute to vascular disease; indeed, this interaction leads to structural modification and functional alteration of the extracellular matrix proteins, promote the generation of reactive oxygen species (ROS) and consequently activates mitogen-activated protein kinase (MAPK) and nuclear factor kappa-B (NF-κB) signaling, followed by production of several inflammatory and/or profibrotic factors which are involved in the progression of atherosclerosis [1].

sRAGE is found to be negatively associated with baseline inflammation [2] but its role in development of cardiovascular diseases and organ damage (OD) is still controversial.

In previous studies, lower levels of sRAGE were found in metabolic disorders and vascular diseases (hypertension, coronary artery disease and peripheral artery disease) when compared to healthy controls [1–6]; conversely it has been reported that sRAGE are elevated in type 1 and type 2 diabetes, in patients with renal impairment [7] and were associated with incident events in a cardiovascular disease population [8].

Hypertension is one of the major risk factors in the development of atherosclerosis and vascular events including stroke, coronary artery disease and peripheral artery disease [9, 10]. Subclinical organ damage is detectable at cardiac site and various vascular districts focusing various parameters including coronary calcifications, carotid intima-media thickness, microalbuminuria, retinal vascular changes and is associate with increased cardiovascular risk in hypertension [11, 12]. In particular, the cardiac chamber morpho-functional changes are early signs of potentially evolving ouvert cardiac failure and atrial fibrillation and ultrasound evaluation allows to identify individuals at high risk due to presence of subclinical cardiac alterations in front of mild to moderate BP elevations [13].

The increasing emphasis on early prevention [9, 10] has prompt the need to focus on patient populations presenting at the early stages of hypertension. Despite the finding of altered sRAGE in high cardiovascular risk populations, there are very few data about the correlation between sRAGE and hypertension disease, in particular it has not been shown whether sRAGE may be reduced in the very early stage of hypertension-associated cardiac damage. Therefore, we sought to determine the sRAGE values in otherwise healthy patients with mildly increased blood pressure (BP) levels and, as a secondary endpoint, evaluate their association with both BP values and metabolic parameters, and with subclinical initial signs of cardiac target organ damage (TOD).

## Methods

We studied 100 consecutive hypertensive patients referred to our hypertension outpatient clinic (Department of Medicine and Surgery, University of Insubria, Varese, Italy) by general practitioners from October 2016 to September 2017, with office blood pressure ≥ 140/90 mmHg and 24-hour (24-h) ambulatory blood pressure monitoring (ABPM) values ≥ 130/80 mmHg [9]. Moreover, 100 subjects with BP < 140/90 mmHg and 24-h ABPM values < 130/80 mmHg were evaluated for a clinical check up and were matched for age, gender and BMI (to avoid possible confounding factors such as overweight and obesity) and enrolled in the study as normotensive controls. As for inclusion criteria, all the patients presented without overt cardiovascular diseases, diabetes and chronic kidney disease, were not smokers and with no history of chronic obstructive pulmonary disease or obstructive sleep apnoea, and were never treated with antihypertensive drugs or statins. Subjects with office systolic BP > 160 mmHg and/or diastolic BP > 100 mmHg and/or body mass index (BMI) ≥ 35 kg/m^2^, subjects who did not show a good window for echocardiographic examination and patients with secondary forms of hypertension were excluded by this study.

All the subjects gave an informed consent to the study which was approved by the local Ethics Committee.

All the subjects underwent a clinic visit including clinical history, anthropometric parameters examination, BMI and waist circumference (cm). Metabolic syndrome was defined according to AHA criteria [14]. Office BP was considered the mean of at least three recordings taken at the time of first visit [9]. All the subjects were submitted to 24-h ABPM, to central BP evaluation, to an echocardiographic examination and to laboratory testing.

### ABPM

ABPM was performed on the non-dominant arm using the Spacelabs Healthcare 90207 (Issaquah, WA, USA). The device was programmed to obtain BP readings at 15-min intervals during the whole recording. The recording was then analyzed in order to obtain 24 h, daytime and night-time average systolic, diastolic blood pressure and heart rate. In 12 subjects the valid measurements were < 70% and the subjects repeated the monitoring within 1 week. As for inclusion criteria, all hypertensive patients showed values > 130/80 mmHg and normotensives showed values below 130/80 mmHg [9].

### Ultrasound examination

Every echocardiographic examination was performed by a single operator by Esaote My-Lab 30 CV ultrasound system (Esaote, Firenze, Italy) using a 2–4 MHz transducer. Examiner was blinded to the results of BP and blood tests. Left ventricle (LV) end-diastolic diameter (LVEDD), LV posterior wall thickness (PWT), and interventricular septum wall thickness (ISWT) were obtained by M-Mode analysis and LV end-diastolic volume (LVEDV) was determined according to the recommendations of the European Association of Echocardiography/American Society of Echocardiography [15]. Systolic function parameters such as ejection fraction (EF), mid-wall-fractional-shortening (MWFS) were estimated using the over cited recommendations. Relative wall thickness (RWT) was calculated and concentric geometry was defined as a RWT > 0.42. The left ventricular mass (LVM) was calculated [15] and indexed for body surface area (LVM/BSA) and for height^2.7^. LV hypertrophy was defined as left ventricular mass index (LVMi: LVM/Height^2.7^) > 48 g/m^2.7^ for men and > 44 g/m^2.7^ for women.

LV diastolic function analysis required information derived from trans-mitral and myocardial Tissue Doppler analysis (TDI). LV diastolic function analysis included the measurement of transmitral early diastolic peak flow velocity (E), the late diastolic flow velocity (A), their ratio (E/A). TDI was used to obtain the LV myocardial longitudinal velocities in the apical four-chamber view with a 2-mm sample volume placed on the basal segment of postero-septal, antero-lateral, inferior and anterior segment during diastole (E′/A′). The (E/E′) ratio of the LV was determined using a mean of E′ obtained between septal and lateral values [22]. Left Atrium diameter (LAD, mm) was measured using the modified Simpson’s method. Maximum left atrium areas, except for the confluence of pulmonary veins and the left atrial appendage, were traced in apical 2- and 4-chamber (4C) views at end systole of the LV (LAV, ml). Left atrial volume-4 chamber-LAV-4C was indexed for body surface area (LAVi, ml/m^2^) and was left atrial volume was considered to be increased if LAVi > 34 ml/m^2^. The intraobserver and interobserver variabilities in our laboratory were 4% and 6%, respectively.

Cardiac TOD was defined as the presence of signs of left ventricular hypertrophy: (LVMi > 48 g/m^2.7^ for men and > 44 g/m^2.7^ for women) and/or increased left atrial volume (LAVi > 34 ml/m^2^).

### Routine laboratory examination

Every patient underwent blood and urinary tests including white blood cell and neutrophil count, C-reactive protein (CRP), lipid profile (total, low-density and high-density cholesterol, triglycerides), fasting glucose, microalbuminuria. The homeostasis model assessment of insulin resistance index (HOMA-IR) was calculated [16]. The glomerular filtration rate (GFR) was calculated according to the modification of diet in renal disease formula < 45 ml/min/1.73 m^2^ [17].

### Laboratory examination of sRAGE

In patients and controls, blood samples were taken in ethylenediaminetetraacetic acid (EDTA) containing tubes after a 14-h overnight fasting for sRAGE quantification. Blood samples were centrifuged at 1000*g* for 30 min and immediately divided into aliquots. Plasma specimens were then frozen and stored at − 20 °C until analysis (which were performed at the Molecular Medicine Research Center, University of Pavia, Pavia, Italy). Plasma sRAGE levels were determined using a commercially available enzyme-linked immunosorbent assay kit (Quantikine; R&D systems) according to the manufacturer’s protocol [14]. Briefly, a monoclonal antibody against sRAGE was used to capture sRAGE from plasma. Captured sRAGE was detected with a polyclonal antihuman sRAGE antibody. After washing, plates were incubated with streptavidin-HRP, developed with appropriate substrate, and OD450 was determined using an enzyme-linked immunosorbent assay plate reader. The intra-assay and inter-assay coefficients of variation values were < 6% and < 8%, respectively. Measurements were performed in duplicate and the results were averaged.

### Statistical analysis

Statistical analysis was performed using the SPSS package for Windows (version 18, Chicago, Illinois, USA). Continuous variables were presented as mean ± SD and were compared using *t* test for two independent samples as they showed a normal distribution. Differences in proportions were compared using the chi2-test. Pearson’s correlation coefficient was used for determining the correlation between different parameters. In the case of non normal distribution of variables, data are reported as median and interquartile range (IQR) and comparisons were made using Mann–Whitney-U test and Spearman correlation analysis.

sRAGE were also evaluated according to the presence or absence of cardiac TOD in hypertensive patients, using ANOVA with LSD post hoc test. In hypertensives, a multivariable general linear regression model was fitted to investigate whether sRAGE was independently associated with subclinical cardiac TOD (LVMi and LAVi), while adjusting for 24-h BP parameters (24-h systolic BP), age, BMI, and HOMA-IR.

A 2-sided p-value of less than 0.05 was considered statistically significant.

## Results

The hypertensive patients and normal subjects had similar age (45.5 ± 6.3 vs. 45.5 ± 6.0, p = 0.767, respectively), sex distribution (male sex: 46% vs 38%, p = 0.315, respectively), BMI (25.9 ± 4.2 kg/m^2^ vs 25.8 ± 4.2 kg/m^2^, p = 0.558, respectively) and waist circumferences (males: 96.2 ± 7.3 cm vs 93.3 ± 8.8, p = 0.140; females: 88.6 ± 10.8 vs 89.5 ± 11, p = 0.666, in hypertensive and normotensive subjects, respectively). The metabolic syndrome was observed in 23% of hypertensive and 7% of normotensive subjects (p = 0.003).

The office, 24-h BP values and arterial tonometry parameters are detailed in Table 1.

**Table 1:** Office, 24-h BP values and arterial tonometry parameters in hypertensive and normotensive subjects

	Hypertensive patients (n = 100)	Normotensive subjects (n = 100)	p
Office SBP (mmHg)	136.5 ± 14.3	120.3 ± 9.6	< 0.001
Office DBP (mmHg)	89.1 ± 8.9	76.1 ± 7.1	< 0.001
Office PP (mmHg)	47.4 ± 8.6	44.2 ± 7.7	0.001
Office heart rate (beats/min)	72.8 ± 10.7	69.4 ± 10.1	0.012
24-h SBP (mmHg)	131.3 ± 7.9	116.9 ± 6.6	< 0.001
24-h DBP (mmHg)	85.3 ± 5.5	72.8 ± 5.1	<0.001
24-h PP (mmHg)	46.0 ± 6.6	44.0 ± 5.6	0.004
Daytime SBP (mmHg)	136.9 ± 8.6	122.7 ± 7.4	< 0.001
Daytime DBP (mmHg)	90.5 ± .5.9	78.2 ± 5.8	< 0.001
Daytime PP (mmHg)	46.3 ± 6.8	44.4 ± 6.2	0.006
Nighttime SBP (mmHg)	120.9 ± 8.8	106.9 ± 7.7	< 0.001
Nighttime DBP (mmHg)	75.7 ± 7.3	63.6 ± 5.7	< 0.001
Nighttime PP (mmHg)	45.2 ± 6.7	43.3 ± 6.5	0.014
24-h HR (beats/min)	74.5 ± 8.7	71.1 ± 8.3	0.010
Daytime HR (beats/min)	79.3 ± 9.7	75.9 ± 9.3	0.023
Nighttime HR (beats/min)	66.5 ± 8.2	63.6 ± 7.8	0.020

*SBP* systolic blood pressure, *DBP* diastolic blood pressure, *PP* pulse pressure, *HR* heart rate

At standard laboratory findings, fasting glucose (93.7 ± 10.2 and 94 ± 9.1 mg/dl, in hypertensives and normotensives, respectively; p = 0.846) and GFR (81 ± 12 vs 83.9 ± 12.4 ml/min/1.73 m^2^ p = 0.479, in hypertensives and normotensives, respectively) were similar in the two groups whereas HOMA-IR [2.8 (1.5–4.9) vs 2.24 (1.1–3.36); p = 0.005] and microalbuminuria [0.8 (0.4–1.4) vs 0.6 (0.3–0.9); p = 0.008] were higher in hypertensive patients. The circulating white blood cells, lipid parameters (except for triglycerides) and CRP values did not differ between hypertensive and normotensive subjects (see Additional file 1: Table S1).

### Echocardiographic parameters in hypertensive and normotensive subjects

Although the morphological and functional properties of left atrium and left ventricle were found within the normal ranges in most of mild hypertensive and normotensive subjects, the interventricular septum, posterior wall thickness and left ventricular mass indexed for BSA were increased in mild hypertensives, whereas the mid-wall-fractional-shortening and the E/A were reduced the hypertensive group; the left atrium parameters were similar between hypertensive and normotensive subjects. Echocardiographic parameters are detailed in Table 2.

**Table 2:** Echocardiographic parameters in hypertensive and normotensive subjects

	Hypertensive patients (n = 100)	Normotensive subjects (n = 100)	p
ISWT (mm)	10.0 ± 1.5	9.2 ± 1.3	0.001
PWT (mm)	8.8 ± 1.2	8.2 ± 1.5	0.001
LVEDD (mm)	45.5 ± 4.3	45.9 ± 4.8	0.440
LVEDV (ml)	94.4 ± 25.6	92.0 ± 27.0	0.698
LVM (g)	146.1 ± 38.4	133.1 ± 35.6	0.056
LVM/BSA (g/m^2^)	78.6 ± 15.5	72.9 ± 15.5	0.017
LVMi (g/m^2.7^)	35.0 ± 7.3	32.8 ± 7.4	0.065
RWT	0.40 ± 0.1	0.36 ± 0.1	< 0.001
EF (%)	63.1 ± 4.9	63.6 ± 5.3	0.984
MWFS	17.0 ± 3.7	19.5 ± .4.6	< 0.001
E/A	1.1 ± 0.3	1.3 ± 0.6	0.028
Septum E′/A′	0.9 ± 0.3	1.1 ± 0.4	< 0.001
Septum E/E′	7.2 ± 1.6	7.2 ± 5.6	0.993
Lateral E′/A′	1.3 ± 0.5	1.5 ± 0.5	0.094
Lateral E/E′	5.1 ± 1.4	5.1 ± 1.4	0.651
LAD (mm)	33.1 ± 4.1	33.2 ± 4.1	0.710
LAV 4C (ml)	40.4 ± 12.8	38.9 ± 13.7	0.479
LAVi (ml/m^2^)	21.9 ± 6.2	21.1 ± 6.9	0.507

*ISWT* interventricular septum wall thickness, *PWT* posterior wall thickness, *LVEDD* left ventricle end-diastolic diameter, *LVEDV* left ventricle end-diastolic volume, *LVM* left ventricle mass, *LVM/BSA* left ventricle mass indexed for body surface area, *LVMi* left ventricular mass index (i.e. LVM indexed for height: LVM/Height^2.7^), *RWT* relative wall thickness, *EF* ejection fraction, *MWFS* mid-wall-fractional-shortening, *E* transmitral early diastolic peak flow velocity, *A* late diastolic flow velocity and E’ and A’ are the parameters obtained by Tissue Doppler analysis, *LAD* left atrium diameter, *LAV 4C* left atrial volume-4 chamber, *LAVi* left atrial volume-4 chamber indexed for body surface area

When the hypertensive patients were subgrouped according to the presence (Hyp-TOD) or absence (Hyp-withoutTOD) of early signs of cardiac TOD,  fifteen subjects showed left ventricular hypertrophy and/or left atrium enlargement. In particular, LVMi was in Hyp-TOD: 42.878 ± 9.456 g/m^2.7^ vs. in Hyp-withoutTOD: 33.259 ± 5.733 g/m^2.7^ (p = 0.002), and LAVi: 28.855 ml/m^2^ vs. 20.632 ± 4.884 ml/m^2^ (p < 0.001).

### sRAGE in hypertensives with and without echocardiographic signs of subclinical TOD and normotensive subjects

**Fig. 1:**
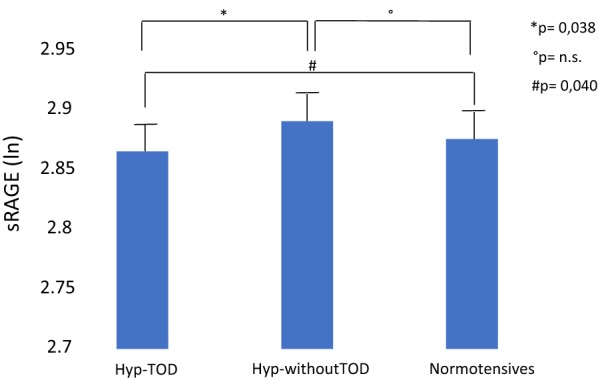
sRAGE in normotensive subjects and in hypertensive patients with or without target organ damage. Lower sRAGE levels were observed in hypertensive patients with target organ damage (Hyp-TOD) when compared with hypertensives without target organ damage (Hyp-withoutTOD) (p = 0.038) and with normotensive subjects (p = 0.040). Columns show plasma levels of sRAGE in: hypertensive patients with cardiac target organ damage (Hyp-TOD), hypertensive patients without cardiac target organ damage (Hyp-without TOD), normotensive subjects. Plasma sRAGE concentration is expressed in natural logarithm. Bars show standard deviations of the three values. *Means statistical significance of the sRAGE levels between Hyp-TOD and Hyp-without TOD. °Means statistical significance of the sRAGE levels between Hyp-without TOD and Normotensives. ^#^Means statistical significance of the sRAGE levels between Hyp-TOD and normotensives

sRAGE levels were similar between hypertensive and normotensive subjects (2.891 ± 0.259 ln-sRAGE vs 2.876 ± 0.263 ln-sRAGE p = 0.691, respectively). However, when subgrouping the hypertensive patients in Hyp-TOD and Hyp-withoutTOD, sRAGE was found to be different among the three groups (p = 0.030), being lower in the Hyp-TOD group than the values of both Hyp-withoutTOD (p = 0.038) and normotensives (p = 0.040) (Fig. 1).

**Fig. 2:**
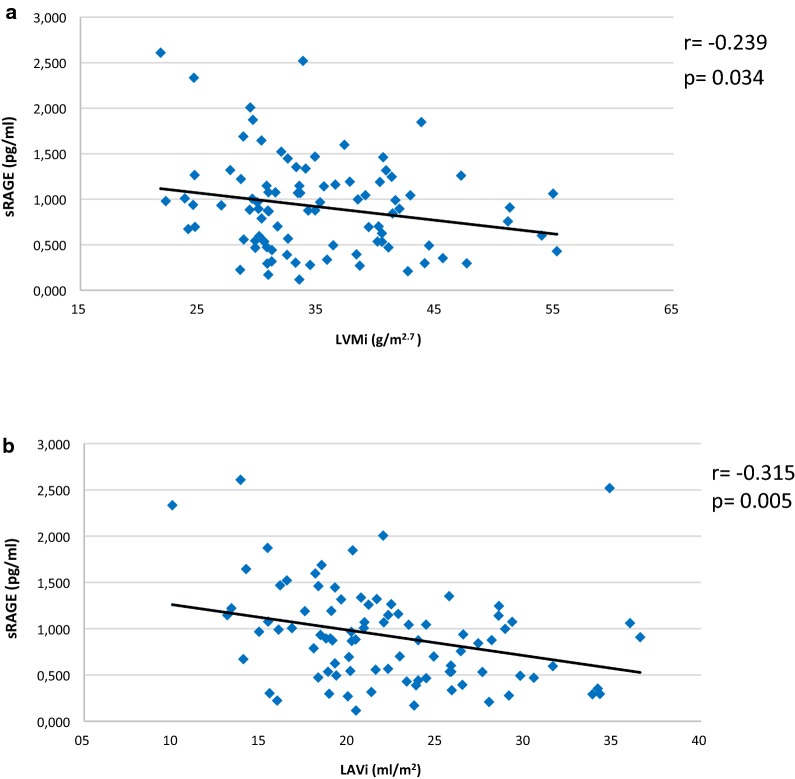
Relationship between sRAGE and left ventricular mass index and left atrial volume index in hypertensives. Figure 2 shows, in the hypertensive group, the relationship between sRAGE and left ventricular mass index (LVMi) (left ventricular mass indexed for height-g/m^2.7^-) (**a**) and left atrial volume index (LAVi) (left atrial volume-4 chamber-indexed for body surface area-ml/m^2^-) (**b**)

sRAGE was not significantly correlated with office and 24-h BPs values (sRAGE and office BPs: r = 0.032, p = 0.768 and r = 0.019, p = 0.864 for systolic and diastolic BP, respectively; sRAGE and 24-h BPs: r = 0.028, p = 0.794 and r = 0.048, p = 0.654 for 24-h systolic and diastolic BPs, respectively). In hypertensives (Hyp-TOD + Hyp-withoutTOD), sRAGE was negatively related with both LVMi (r = − 0.239, p = 0.034) and LAVi (r = − 0.315, p = 0.005) (Fig. 2a, b, respectively).

In multivariable analysis, in hypertensive group (Hyp-TOD + Hyp-withoutTOD), when entering sRAGE as dependent variable, and subclinical cardiac TOD, 24-h systolic BP, mean age, BMI, and HOMA-IR as independent variables, a significant association was observed (model p for ANOVA p = 0.019, R = 0.354, R square = 0.100). The association was significant for TOD (β = − 0.233, p = 0.04) whereas the other variables did not fit the model (24-h systolic BP: β = − 0.022, p = 0.844; mean age: β = 0.152, p = 0.197; BMI: β = − 0.050, p = 0.659; and HOMA-IR: β = − 0.188, p = 0.107).

## Discussion

### Main results

To our knowledge this is the first study exploring the relationship between circulating sRAGE and subclinical cardiac TOD in otherwise healthy, untreated patients with a recent diagnosis of mild hypertension. As expected according to inclusion and exclusion criteria, mean morphological and functional parameters of left cardiac chambers were found within the normal ranges in most of the patients in both the mild hypertensives and the normotensive subjects. Among the group of patients at the early stage of the hypertensive disease, fifteen patients showed early echographic signs of cardiac TOD, being LVMi and/or LAVi increased above the normal ranges. Although sRAGE were similar between mild hypertensives and normotensives, and not related to either office or 24-h BP values, when subgrouping the population of mild untreated hypertensives according to the presence or absence of TOD, reduced sRAGE levels were observed in the Hyper-TOD group with early alteration in cardiac parameters.

Although only fifteen patients had LVMi and/or LAVi increased above the normal ranges, the correlation between sRAGE and subclinical cardiac TOD was confirmed also in the multivariable analysis.

### sRAGE and hypertension organ damage (OD)

Our study report the association of sRAGE with very early left ventricle and left atrium modification, in a relatively young (with ages within a relatively narrow range) and otherwise healthy mild hypertensives, suggesting that decreased sRAGE levels may occur very early in the development of the hypertensive disease.

In literature there are no univocal results about the role of sRAGE in cardiovascular disease, beyond that the origin and progression of OD.

Previously, sRAGE decreased plasma levels were described in a population of hypertensive patients including severe stages of hypertension [5] and left ventricular hypertrophy (indexed for body surface area) correlated inversely with sRAGE in a group of long-lasting treated hypertensive disease [18]. As confounding factors, the latter study included older patients among the 81 presenting with left ventricular hypertrophy and a large percentage of patients with coronary artery disease [25]. A relationship between sRAGE and left ventricular hypertrophy was also reported in patients with chronic kidney disease with severely impaired GFR [19].

Decreased sRAGE levels were also found to be associated with high carotid intima-media thickness (IMT) of the common carotid artery and with carotid plaque calcifications in 284 subjects without history of atherosclerosis disease, suggesting a role of sRAGE in vascular inflammation and in early-stage atherosclerosis [20]. This hypothesis is supported by the evidence that sRAGE may be implicated in impaired glucose metabolism in patients with primary hypertension [21].

Conversely, Heier et al. showed in a 5 years follow up study a possible protective effect of high levels of sRAGE against inflammation but not for carotid atherosclerosis in a cohort of adolescents and young adults with diabetes type 1 [22]. Moreover, Paradela-Dobarro et al. showed that while AGEs and sRAGE are bad prognostic biomarkers for acute heart failure (HF), they serve as useful markers of HF progression, suggesting the role of the axis AGE-RAGE activation in cardiac organ damage [23].

### sRAGE, oxidative stress and inflammation

Circulating sRAGE values have been negatively associated to inflammation in humans. Indeed, low sRAGE levels have been associated with elevated circulating inflammatory markers such as CRP and white blood cells in large patient populations [2, 4, 22].

Oxidative stress and autophagy were found to be related to both cardiac hypertrophy and heart failure in a distinct manner in animal models [24]. Myocyte hypertrophy induced in rats after pressure overload was the result of autophagy impairment induced by oxidative stress [25]. Moreover AGEs can cause endothelial dysfunction via decreased endothelial nitric oxide synthase expression and increased oxidative stress in human coronary artery endothelial cells by activation of the mitogen-activated protein kinases p38 and ERK1/2 [26].

In our patients with newly diagnosed and mild stage of hypertension, the finding of sRAGE reduction in the subgroup of patients presenting with initial cardiac TOD, although the small number size, may supports the hypothesis that reduced sRAGE may be an early metabolic sign related to subclinical anatomical cardiac remodeling in hypertension, as a marker of increased oxidative stress, even though do not directly supported by our data.

Although sRAGE was not associated with incidence of atrial fibrillation in the AIRC study [2], it has been demonstrated that oxidative stress and inflammation may affect adverse atrial structural and electrical remodeling that lead to the onset and maintenance of atrial fibrillation [27] and high sRAGE were associated with favorable clinical outcome after catheter ablation in diabetic patients [28]. Moreover, when stratified according to glycated hemoglobin (HbA1c), patients with prediabetes (HbA1c 5.7% to 6.4%) exhibited subclinical atrial alterations associated with sRAGE [29].

It has been suggested in animal models that antioxidant treatment could have a role in prevention of early myocardial hypertrophy [25]. It is well known that drugs such as statins may interfere with the pro-inflammatory immune cell attitude through Angiotensin II AT1 receptor expression modulation [30–32] and statin have been shown to be associated with decreased incident atrial fibrillation episodes after cardioversion [33]. Moreover, various cardiovascular drugs may modify advanced-glycation-end-products and sRAGE levels [34, 35]. In our study no patients had suffered episodes of atrial fibrillation or was on therapy with anti-inflammatory drugs or antihypertensives or statins which could have influenced the oxidative status or morphofunctional atrial changes of the patients.

Therefore, in this patient population of untreated and mild, recently diagnosed hypertensive patients, low circulating sRAGE may be a very early marker of initial TOD.

## Additional file

**Additional file 1: Table S1.** Circulating white blood cells and lipid parameters in hypertensive and normotensive subjects.

## Abbreviations

A: late diastolic flow velocity
ABPM: ambulatory blood pressure monitoring
AHA: American Heart Association
BMI: body mass index
BP: blood pressure
BSA: body surface area
CRP: C-reactive protein
DBP: diastolic blood pressure
E: transmitral early diastolic peak flow velocity
EDTA: ethylenediaminetetraacetic acid
EF: ejection fraction
GFR: glomerular filtration rate
HbA1c: glycated hemoglobin
HDL: high density lipoprotein
HF: heart failure
HOMA-IR: homeostasis model assessment of insulin resistance index
HR: heart rate
IMT: intima-media thickness
IQR: interquartile range
ISWT: interventricular septum wall thickness
LAD: left atrium diameter
LAV: left atrial volume
LAVi: left atrial volume 4-chamber indexed for body surface area
LDL: low density lipoprotein
LVEDD: left ventricle end-diastolic diameter
LVEDV: left ventricle end-diastolic volume
LVH: left ventricular hypertrophy
LVM: left ventricular mass
LVMi: left ventricular mass index
MWFS: mid-wall-fractional-shortening
OD: organ damage
PP: pulse pressure
PWT: posterior wall thickness
RWT: relative wall thickness
SBP: systolic blood pressure
SD: standard deviation
sRAGE: soluble Receptor for Advanced Glycation End products
TDI: tissue doppler analysis
TOD: target organ damage
4C: 4-chamber

## References

[1] Fukami K, Yamagishi S, Okuda S (2014). Role of AGEs-RAGE system in cardiovascular disease. Curr Pharm Des.

[2] Al Rifai M, Schneider AL, Alonso A, Maruthur N, Parrinello CM, Astor BC, Hoogeveen RC, Soliman EZ, Chen LY, Ballantyne CM, Halushka MK, Selvin E (2015). sRAGE, inflammation, and risk of atrial fibrillation: results from the Atherosclerosis Risk in Communities (ARIC) Study. J Diabetes Complications.

[3] Falcone C, Emanuele E, D’Angelo A (2005). Plasma levels of soluble receptor for advanced glycation end products and coronary artery disease in nondiabetic men. Arterioscler Thromb Vasc Biol.

[4] Falcone C, Buzzi MP, Bozzini S, TALENTInvestigators (2013). Relationship between sRAGE and eotaxin-3 with CRP in hypertensive patients at high cardiovascular risk. J Nephrol.

[5] Geroldi D, Falcone C, Emanuele E, D’Angelo A, Calcagnino M, Buzzi MP, Scioli GA, Fogari R (2005). Decreased plasma levels of soluble receptor for advanced glycation end-products in patients with essential hypertension. J Hypertens.

[6] Falcone C, Bozzini S, Guasti L, D’Angelo A, Capettini AC, Paganini EM, Falcone R, Moia R, Gazzaruso C, Pelissero G (2013). Soluble RAGE plasma levels in patients with coronary artery disease and peripheral artery disease. Sci World J.

[7] Prasad K (2014). Low levels of serum soluble receptors for advanced glycation end products, biomarkers for disease state: myth or reality. Int J Angiol.

[8] Reichert S, Triebert U, Santos AN, Hofmann B, Schaller HG, Schlitt A, Schulz S (2017). Soluble form of receptor for advanced glycation end products and incidence of new cardiovascular events among patients with cardiovascular disease. Atherosclerosis..

[9] Mancia G, Fagard R, Narkiewicz K, Redón J, Zanchetti A, Böhm M, Christiaens T, Cifkova R, De Backer G, Dominiczak A, Galderisi M, Grobbee DE, Jaarsma T, Kirchhof P, Kjeldsen SE, Laurent S, Manolis AJ, Nilsson PM, Ruilope LM, Schmieder RE, Sirnes PA, Sleight P, Viigimaa M, Waeber B, Zannad F (2013). Task Force Members 2013 ESH/ESC Guidelines for the management of arterial hypertension: the Task Force for the management of arterial hypertension of the European Society of Hypertension (ESH) and of the European Society of Cardiology (ESC). J Hypertens.

[10] Whelton PK, Carey RM, Aronow WS, Casey DE, Collins KJ, Dennison Himmelfarb C, DePalma SM, Gidding S, Jamerson KA, Jones DW, MacLaughlin EJ, Muntner P, Ovbiagele B, Smith SC, Spencer CC, Stafford RS, Taler SJ, Thomas RJ, Williams KA, Williamson JD, Wright JT (2017). 2017ACC/AHA/AAPA/ABC/ACPM/AGS/APhA/ASH/ASPC/NMA/PCNA Guideline for the Prevention, Detection, Evaluation, and Management of High Blood Pressure in Adults: A Report of the American College of Cardiology/American Heart Association Task Force on Clinical Practice Guidelines. Hypertension..

[11] Cuspidi C, Valerio C, Sala C, Esposito A, Masaidi M, Negri F, Zanchetti A, Mancia G (2008). Prevalence and correlates of multiple organ damage in a never-treated hypertensive population: role of ambulatory blood pressure. Blood Press Monit..

[12] Lehmann N, Erbel R, Mahabadi AA, Kälsch H, Möhlenkamp S, Moebus S, Stang A, Roggenbuck U, Strucksberg KH, Führer-Sakel D, Dragano N, Budde T, Seibel R, Grönemeyer D, Jöckel KH, Heinz Nixdorf Recall Study Investigators (2016). Accelerated progression of coronary artery calcification in hypertension but also prehypertension. J Hypertens..

[13] Cuspidi C, Tadic M, Sala C, Grassi G (2015). How to identify hypertensive patients at high cardiovascular risk? The role of echocardiography. High Blood Press Cardiovasc Prev..

[14] Grundy SM, Cleeman JI, Daniels SR, Donato KA, Eckel RH, Franklin BA, Gordon DJ, Krauss RM, Savage PJ, Smith SC, Spertus JA, Costa F, American Heart Association; National Heart, Lung, and Blood Institute (2005). Diagnosis and management of the metabolic syndrome: an American Heart Association/National Heart, Lung, and Blood Institute Scientific Statement. Circulation..

[15] Lang RM, Badano LP, Mor-Avi V, Afilalo J, Armstrong A, Ernande L, Flachskampf FA, Foster E, Goldstein SA, Kuznetsova T, Lancellotti P, Muraru D, Picard MH, Rietzschel ER, Rudski L, Spencer KT, Tsang W, Voigt JU (2015). Recommendations for cardiac chamber quantification by echocardiography in adults: an update from the American Society of Echocardiography and the European Association of Cardiovascular Imaging. J Am Soc Echocardiogr..

[16] Matthews DR, Hosker JP, Rudenski AS, Naylor BA, Treacher DF, Turner RC (1985). Homeostasis model assessment: insulin resistance and beta-cell function from fasting plasma glucose and insulin concentrations in man. Diabetologia.

[17] Klahr S, Levey AS, Beck GJ (1994). For the Modification of Diet in Renal Disease Study Group: the effects of dietary protein restriction and blood pressure control on the progression of chronic renal disease. N Engl J Med.

[18] Liu Q, Chen HB, Luo M, Zheng H (2016). Serum soluble RAGE level inversely correlates with left ventricular hypertrophy in essential hypertension patients. Genet Mol Res..

[19] Leonardis D, Basta G, Mallamaci F, Cutrupi S, Pizzini P, Tripepi R, Tripepi G, De Caterina R, Zoccali C (2012). Circulating soluble receptor for advanced glycation end product (sRAGE) and left ventricular hypertrophy in patients with chronic kidney disease (CKD). Nutr Metab Cardiovasc Dis..

[20] Moriya S, Yamazaki M, Murakami H, Maruyama K, Uchiyama S (2014). Two soluble isoforms of receptors for advanced glycation end products (RAGE) in carotid atherosclerosis: the difference of soluble and endogenous secretory RAGE. J Stroke Cerebrovasc Dis..

[21] Wang Y, Zhang W, Zhao H, Wang Y, Lu C, Li X, Wang Y, Xiao Y, Wang Y, Wang B (2018). Fasting blood soluble RAGE may be causally implicated in impaired glucose metabolism in Chinese patients with primary hypertension. Gene.

[22] Heier M, Margeirsdottir HD, Gaarder M, Stensæth KH, Brunborg C, Torjesen PA, Seljeflot I, Hanssen KF, Dahl-Jørgensen K (2015). Soluble RAGE and atherosclerosis in youth with type 1 diabetes: a 5-year follow-up study. Cardiovasc Diabetol..

[23] Paradela-Dobarro B, Fernández-Trasancos Á, Bou-Teen D, Eiras S, González-Ferreiro R, Agra RM, Varela-Román A, Castro-Pais AI, Carreira MC, Casanueva FF, Álvarez E, González-Juanatey JR (2017). Evolution and bad prognostic value of advanced glycation end products after acute heart failure: relation with body composition. Cardiovasc Diabetol..

[24] Li B, Chi RF, Qin FZ, Guo XF (2016). Distinct changes of myocyte autophagy during myocardial hypertrophy and heart failure: association with oxidative stress. Exp Physiol.

[25] Wang JP, Chi RF, Wang K, Ma T, Guo XF, Zhang XL, Li B, Qin FZ, Han XB, Fan BA (2018). Oxidative stress impairs myocyte autophagy, resulting in myocyte hypertrophy. Exp Physiol. Exp Physiol..

[26] Ren X, Ren L, Wei Q, Shao H, Chen L, Liu N (2017). Advanced glycation end-products decreases expression of endothelial nitric oxide synthase through oxidative stress in human coronary artery endothelial cells. Cardiovasc Diabetol..

[27] Karam BS, Chavez-Moreno A, Koh W, Akar JG, Akar FG (2017). Oxidative stress and inflammation as central mediators of atrial fibrillation in obesity and diabetes. Cardiovasc Diabetol..

[28] Yang PS, Kim TH, Uhm JS, Park S, Joung B, Lee MH, Pak HN (2016). High plasma level of soluble RAGE is independently associated with a low recurrence of atrial fibrillation after catheter ablation in diabetic patient. Europace..

[29] Di Pino A, Mangiafico S, Urbano F, Scicali R, Scandura S, D’Agate V, Piro S, Tamburino C, Purrello F, Rabuazzo AM (2017). HbA1c identifies subjects with prediabetes and subclinical left ventricular diastolic dysfunction. J Clin Endocrinol Metab.

[30] Marino F, Maresca AM, Cosentino M, Castiglioni L, Rasini E, Mongiardi C, Maio RC, Legnaro M, Schembri L, Dentali F, Grandi AM, Guasti L (2012). Angiotensin II type 1 and type 2 receptor expression in circulating monocytes of diabetic and hypercholesterolemic patients over 3-month rosuvastatin treatment. Cardiovasc Diabetol..

[31] Marino F, Guasti L, Tozzi M, Consuelo Maio R, Castiglioni L, Rasini E, Schembri L, Maroni L, Legnaro M, De Leo A, Piffaretti G, Castelli P, Venco A, Lecchini S, Cosentino M (2009). Angiotensin type 1 receptor expression and interleukin-8 production in polymorphonuclear leukocytes of patients with peripheral arterial disease. J Cardiovasc Pharmacol.

[32] Guasti L, Marino F, Cosentino M, Maio RC, Rasini E, Ferrari M, Castiglioni L, Klersy C, Gaudio G, Grandi AM, Lecchini S, Venco A (2008). Prolonged statin-associated reduction in neutrophil reactive oxygen species and angiotensin II type 1 receptor expression: 1-year follow-up. Eur Heart J.

[33] Dentali F, Gianni M, Squizzato A, Ageno W, Castiglioni L, Maroni L, Hylek EM, Grandi AM, Cazzani E, Venco A, Guasti L (2011). Use of statins and recurrence of atrial fibrillation after catheter ablation or electrical cardio version. A systematic review and meta-analysis. Thromb Haemost.

[34] Falcone C, Buzzi MP, Bozzini S, Boiocchi C, D’Angelo A, Schirinzi S, Esposito C, Torreggiani M, Choi J, Ochan Kilama M, Mancia G (2012). Microalbuminuria and sRAGE in high-risk hypertensive patients treated with nifedipine/telmisartan combination treatment: a substudy of TALENT. Mediators Inflamm.

[35] Prasad K, Tiwari S (2017). Therapeutic interventions for advanced glycation-end products and its receptor- mediated cardiovascular disease. Curr Pharm Des.

