# Supplementary material for: sRAGE and early signs of cardiac target organ damage in mild hypertensives
Source: Cardiovasc Diabetol. 2019 Feb 12;18:17. doi: 10.1186/s12933-019-0821-5 (PMC6371567; doi:10.1186/s12933-019-0821-5)
Supplement: Supplementary file 1 — Additional file 1: Table S1. Circulating white blood cells and lipid parameters in hypertensive and normotensive subjects. [file 12933_2019_821_MOESM1_ESM.docx]

**Table S1. Circulating white blood cells and lipid parameters in hypertensive and normotensive subjects.**

|  | Hypertensive patients  (n=100) | Normotensive subjects  (n=100) | p |
| --- | --- | --- | --- |
| Neutrophils  (x1000/mm^3^) | 3.38±1.38 | 3.37±1.25 | n.s. |
| Lymphocytes  (x1000/mm^3^) | 2.0±0.59 | 2.15±0.56 | n.s. |
| C-reactive protein (mg/dl) | 1 (0.6;2.1) | 1.2 (0.52;2.1) | n.s. |
| Total cholesterol (mg/dl) | 217 (196;236) | 218 (192;243) | n.s. |
| HDL-cholesterol (mg/dl) | 57 (50;68) | 57 (47;67) | n.s. |
| LDL-cholesterol (mg/dl) | 133 (112;153) | 134 (108;156) | n.s. |
| Triglycerides (mg/dl) | 95 (70;119) | 105 (71;157) | 0.02 |

*HDL* high density lipoprotein; *LDL* low density lipoprotein
